# Talking to fewer people leads to having more malleable linguistic representations

**DOI:** 10.1371/journal.pone.0183593

**Published:** 2017-08-24

**Authors:** Shiri Lev-Ari

**Affiliations:** Max Planck Institute for Psycholinguistics, Nijmegen, The Netherlands; University of Edinburgh, UNITED KINGDOM

## Abstract

We learn language from our social environment. In general, the more sources we have, the less informative each of them is, and the less weight we should assign it. If this is the case, people who interact with fewer others should be more susceptible to the influence of each of their interlocutors. This paper tests whether indeed people who interact with fewer other people have more malleable phonological representations. Using a perceptual learning paradigm, this paper shows that individuals who regularly interact with fewer others are more likely to change their boundary between /d/ and /t/ following exposure to an atypical speaker. It further shows that the effect of number of interlocutors is not due to differences in ability to learn the speaker’s speech patterns, but specific to likelihood of generalizing the learned pattern. These results have implications for both language learning and language change, as they suggest that individuals with smaller social networks might play an important role in propagating linguistic changes.

## Introduction

Imagine you only knew two people in the world. One of them owns a hedgehog and the other keeps a skunk as a pet. You should then conclude that hedgehogs and skunks are equally common as pets. If you were to meet a third person who also kept a hedgehog at home, you would have to revise your assumption and conclude that hedgehogs are twice as common pets in the world. But if instead of knowing only two people, you knew 50, with half of them keeping hedgehogs as pets and half of them keeping skunks as pets, meeting another person who owns a hedgehog would have not led you to revise your assumption as drastically. The reason is that there is an inverse relationship between how informative each source is, and how many sources we already have. When we only have three sources, we ascribe each of them a weight that is much higher than what we would ascribe them if we knew fifty one people. The study in this paper tests whether the same logic applies to social network size and linguistic input. In other words, the study here tests whether people with smaller social networks give each speaker greater weight, and are consequently more susceptible to the influence of new speakers.

Past literature has shown that we learn language from the environment by sampling the input in our environment and adjusting our representations in accordance with it. We are sensitive to both the structure of the input and its frequency (e.g., [1–4]). For example, children produce frequent words in their input earlier than less frequent words (e.g.,[1]). Even in adulthood, word frequency facilitates processing (e.g., [5]). Importantly, learning and adaptation of representations continue throughout our lives, leading our representations to be in constant flux. Indeed, examination of our language use throughout the years reveals changes. For example, an analysis of Queen Elisabeth II’s Christmas addresses throughout the years reveals that her pronunciation has drifted with the years leading even the Queen to no longer speak the Queen’s English in the strictest sense [6]. Similarly, exposure to atypical tokens, such as /t/ with a particularly short Voice Onset Time, can lead us to shift our boundary between /t/ and its VOT contrasting phoneme, /d/ (e.g., [7–8]), and even exposure to a new language can lead us to modify our categories in our native language (e.g., [9–13] but see [14]). This process of perceptual learning and adaptation is due to the fact that we adjust our inferences about linguistic boundaries according to the distribution in our input. When the distribution changes, so do our categorical boundaries.

The approach taken in this paper combines exemplar theory [15–16] with Bayesian models of processing, though the main argument and results could fit with other models of language learning and processing as well. The assumption in this paper is that listeners store the tokens they are exposed to together with social information about the speaker. This storage of both acoustic and social information also allows speakers to keep track of the distribution of different features in the input, as well as track multiple distributions that are contingent on speakers’ social characteristics, for instance, that the distributions of vowel formant frequencies differ for men and women. These learnt conditional distributions from the input will determine to which cases we will generalize what we learnt and which conditional distributions will be updated and to what degree (see [17] for a similar account).

Crucially, when learning from our environment we are sensitive not only to the raw frequency but to the number of sources it is derived of. For example, if you were to know that the hedgehog owner that you know had previously owned a different hedgehog which passed away, you would not adjust your representation to conclude that people are twice as likely to own hedgehogs than skunks but realize that sampling the same person twice is not as informative as sampling two different people. Indeed, even chimpanzees normalize frequency by the number of sources, and thus are more likely to copy a behavior when it is performed by two thirds of the demonstrators than when it is performed two thirds of the time, but repeatedly by the same demonstrator [18]. Children are also more likely to copy a behavior when it is displayed by several actors rather than one [19–21]. This suggests that we are also more likely to copy and learn a certain linguistic use when it is used by a higher proportion of sources in our environment.

Understanding speakers’ malleability, and in particular, whether people with smaller social networks have more malleable linguistic representations can have implications for language change. One of the puzzles of language change is how rare variants propagate throughout the community [22]. If people with smaller social networks are more susceptible to the influence of each speaker they encounter, then they might play an important role in propagating linguistic change. This point will be discussed further in the General Discussion. The goal of this study is to first examine whether people with smaller social networks have more malleable linguistic representations.

## Experiment

The aim of the experiment is to test whether individuals with smaller social networks have more malleable linguistic representations. The malleability of individuals' linguistic representations was measured by testing the degree to which their general representations are influenced by perceptual learning. Perceptual learning is the process of adjusting one's representations in response to exposure to (atypical) input. For example, one of the key features that distinguishes /d/ from /t/ is /t/'s longer Voice Onset Time. Exposure to tokens with an intermediate VOT in a context in which those tokens are interpreted as a /d/ or as a /t/ leads to an adjustment of the boundary between the two categories to be at a higher or lower VOT value, respectively (e.g., [7–8]). Importantly, when the variation in production is uninformative about the speaker’s physiology, as is the case with VOT, listeners’ learning is general rather than speaker-specific. That is, listeners adapt their *general* representation of these stop categories following such exposure, as is reflected in an influence on their categorization of stops by a *new* speaker(e.g., [7]).The hypothesis tested in this paper is that having a larger social network would reduce the degree to which listeners would adjust their general representation in response to the atypical input. One potential problem with interpreting such results is that social network size was not experimentally manipulated. Therefore, it could be the case that people with smaller social networks are more motivated to do the task or are better at learning a speaker’s speech pattern. To ensure that the results are not due to such difference, the study also includes a control condition that tests individuals' perception of the stops in the speech of the speaker they had listened to. Unlike the case of a novel speaker, this condition examines whether participants are able to learn the speech patterns of a specific speaker. The hypothesis is that participants' social network size would not influence this ability, as having fewer sources should not influence the ability to learn phonological categories per se, but only the informativity of the input for the wider population. That is, it is hypothesized that social network size does not influence one’s ability to process, store or make inferences according to received input. Therefore, as long as individuals are given the same amount of information about a particular speaker’s production patterns, their social network size should not influence their ability to learn the speaker’s patterns.

### Method

#### Participants

One-hundred-fifty-three native English speakers located in the US participants were recruited via Mechanical Turk (Age: 19–71, M = 37, SD = 12). Participants were asked to only participate if they can use a pair of high quality headphones. Participants received $1.20 for their participation.

#### Ethics statement

The experiment was approved by the ethics board of the Social Sciences Faculty of Radboud University. Participants provided written consent electronically.

#### Stimuli

Social network questionnaire: Participants were asked several questions about their social network, including how many people they orally converse with in a typical week, and how many hours they spend orally conversing with others in a typical week. Participants also answered several additional questions about their social network, such as the interlocutors' educational level. These were included to gather information about people's social network for future research. See Supplementary material for the full questionnaire. Similar versions of this questionnaire have been used in previous studies [23–24]. Responses to the Social Network questionnaire indicated that participants’ network ranged from 1 to 100 (M = 11.11, SD = 11.62). An examination of these responses revealed two participants whose Social Network Size was 4 and 8 standard deviations from the mean. They were therefore excluded, as they would have very high leverage and thus exert undue influence on the regression [25]. After their exclusion, social network size ranged from 1 to 40 (M = 10.18, SD = 8.13). See S1 Text for further details.

Picture selection task: 80 noun phrases of the form "The [adj] [noun]", such as "The yellow toy", were recorded in a soundbooth by one native female speaker of American English. Twenty of the noun phrases included a noun which starts with a /d/, and twenty included a noun that starts with a /t/. These phrases did not include any other stops. 40 additional noun phrases of the same form served as fillers. These did not include any stops. All recordings were paired with a pair of pictures. Both pictures depicted the same object (e.g., a toy) but only one of them fit the description in the noun phrase (e.g., yellow). Participants' task was to click on the picture that fit the description.

Next an alternative version was created for each of the critical noun phrase recordings by replacing the stop (/d/ or /t/) with a token that had a VOT of 24ms. This token was created by excising aspiration out of a natural token of a /t/ in the word *teen*. This token was selected because it is ambiguous between /d/ and /t/. Participants in the manipulated /d/ condition heard all the manipulated /d/ recordings and all the *un*manipulated /t/ recordings, as well as all the filler recordings. The manipulated /t/ condition included all the manipulated /t/ recordings, all the *un*manipualted /d/ recordings, as well as all the filler recordings. The same set of pictures appeared in both conditions.

In both conditions, presentation order was random, with the exception that the first two trials always consisted of filler items.

Phoneme categorization: The same speaker as in the Picture selection task as well as another male native speaker of American English recorded the word *teen*. A continuum from *teen* to *dean* was created for each speaker by excising periods of aspiration out of their original recording of *teen*. The continuum consisted of 5 critical steps, centered around the most ambiguous token (15ms, 20ms, 25ms, 30ms and 35ms), as well as two good exemplars of /d/ (with VOTs of 5 and 10ms), and two good exemplars of /t/ (with VOTs 50 and 80ms). The original recordings of the word *teen* had a VOT of 80ms and 85ms for the old and new speaker, respectively. During the experiment, each critical item repeated 8 times, while each good exemplar item repeated 3 times, for a total of 52 trials. Only responses to the critical steps (15-35ms) were analyzed whereas the good exemplars were included to provide natural anchoring points. The order of appearance was random except for presenting a good exemplar of /t/ in the first trial. Participants' task was to indicate whether the word they heard was *dean* or *teen* by clicking on the boxes with those words on the screen.

#### Procedure

Participants were recruited in November 2014. All participants started by answering the social network questionnaire, followed by the picture selection task, and then the phoneme categorization task. The two exposure conditions (manipulated /d/, manipulated /t/), and the two test conditions (Same Speaker, New Speaker) were manipulated across participants in a fully crossed design, creating 4 between participants conditions. At the end, all participants were asked whether there was anything unusual about the speech of the speaker in the picture selection task, to ensure that the perceptual adjustment was implicit. The entire experiment took between 5 and 15 minutes.

### Results

Three participants commented on the oddity of the pronunciation of /t/s or /d/s in the experiment in the questions at the end of the experiment. They were therefore excluded from all analyses. The analyses were therefore over the remaining 148 participants. An examination of participants’ responses revealed that some of them inappropriately calculated the number of interlocutors. In particular, some participants estimated the number of weekly interlocutors they have to be 6, 9, or even 10 times the number of hours they interact a week. This suggests that they have included people with whom they do not exchange more than a few words, and therefore, from whom they do not receive sufficient input. To ensure that participants' estimates of their number of interlocutors are not inflated, but include only interlocutors from whom they receive sufficient input, participants' estimates for their number of interlocutors per week were trimmed to twice the number of hours they interact per week. See S1 Text for further details.

**Fig 1:**
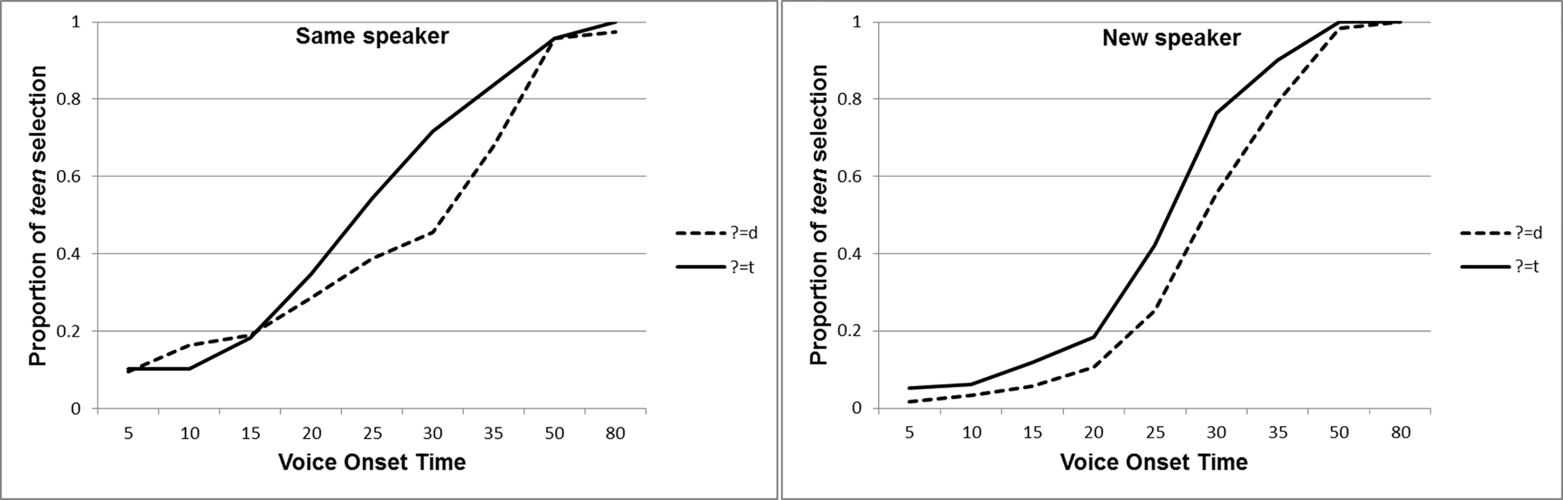
Participants’ phoneme categorization by condition. Participants' proportion of classifying the word as *teen* as dependent on the audio condition (manipulated /d/, manipulated /t/) in the (a) Same Speaker condition and (b) New Speaker condition.

Visual examination of the raw data suggests that the paradigm worked, and that, in general, participants' performance in the phoneme categorization task was influenced by the speech of the speaker in the picture selection task (see Fig 1). To test whether participants with a smaller social network adjusted their representations to a greater degree, a logistic mixed model analysis was conducted in R version 3.2.4 [26] using version 1.1–11 of the lme4 package [27]. The analysis included Participants as a random variable, and VOT (centered around 25), Audio Condition (manipulated /t/, manipulated /d/), Speaker (Same, New), Social Network Size (centered), and the three-way interaction of Social Network Size with Audio Condition and Speaker, including all two-way interactions within, as fixed effects. The model included an intercept for the random variable, and a slope for VOT (This is the only possible slope as all other factors were manipulated between participants). Results revealed an effect of VOT (β = 0.27, SE = 0.01, z = 21.79, p<0.001), such that the higher a token's VOT, the more likely participants were to classify the token as *teen*. As VOT is a key feature that distinguishes /d/ and /t/, this shows that participants were performing the task. Results also revealed an effect of the audio manipulation at the base level, such that participants in the manipulated /t/ condition interpreted more tokens as *teen* (β = 1.06, SE = 0.42, z = 2.51, p = 0.012). This effect confirms that our manipulation worked and participants indeed showed perceptual learning. The effects of Social Network Size (β = 0.08, SE = 0.04, z = 1.90, p = 0.057) and the interaction between Speaker and Social Network Size (β = -0.1, SE = 0.05, z = -1.80, p = 0.073) were in the right direction but did not reach the conventional level of significance. Importantly, these were modulated by a three-way interaction between Speaker, Audio Condition and Social Network Size (β = 0.17, SE = 0.08, z = 2.10, p = 0.036; See S1 Table for the full results).

**Fig 2:**
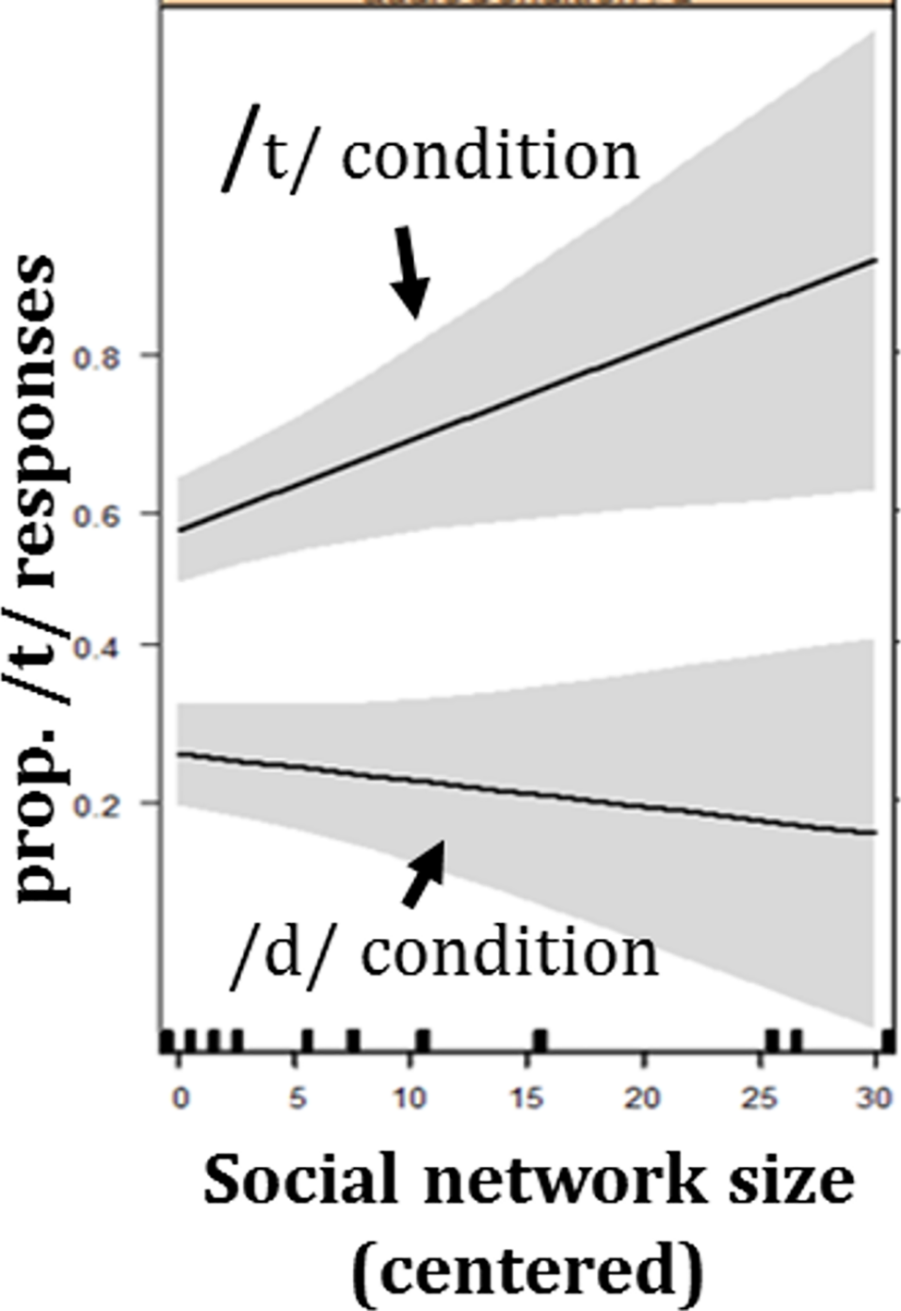
Model’s statistical results for performance in the Same Speaker condition. The model’s predictions for the probability of *teen* selection in the Same Speaker condition as dependent on social Network Size and Audio Condition in exposure. Note that there is *no* interaction between Social Network Size and Audio Condition. The effect of perceptual learning is manifested in the distance between the line in the manipulated /t/ condition and the line in the manipulated /d/ condition, as greater perceptual learning should lead to more *teen* responses in the manipulated /t/ condition and fewer *teen* responses in the manipulated /d/ condition. Gray bands indicate standard errors (67% confidence interval).

**Fig 3:**
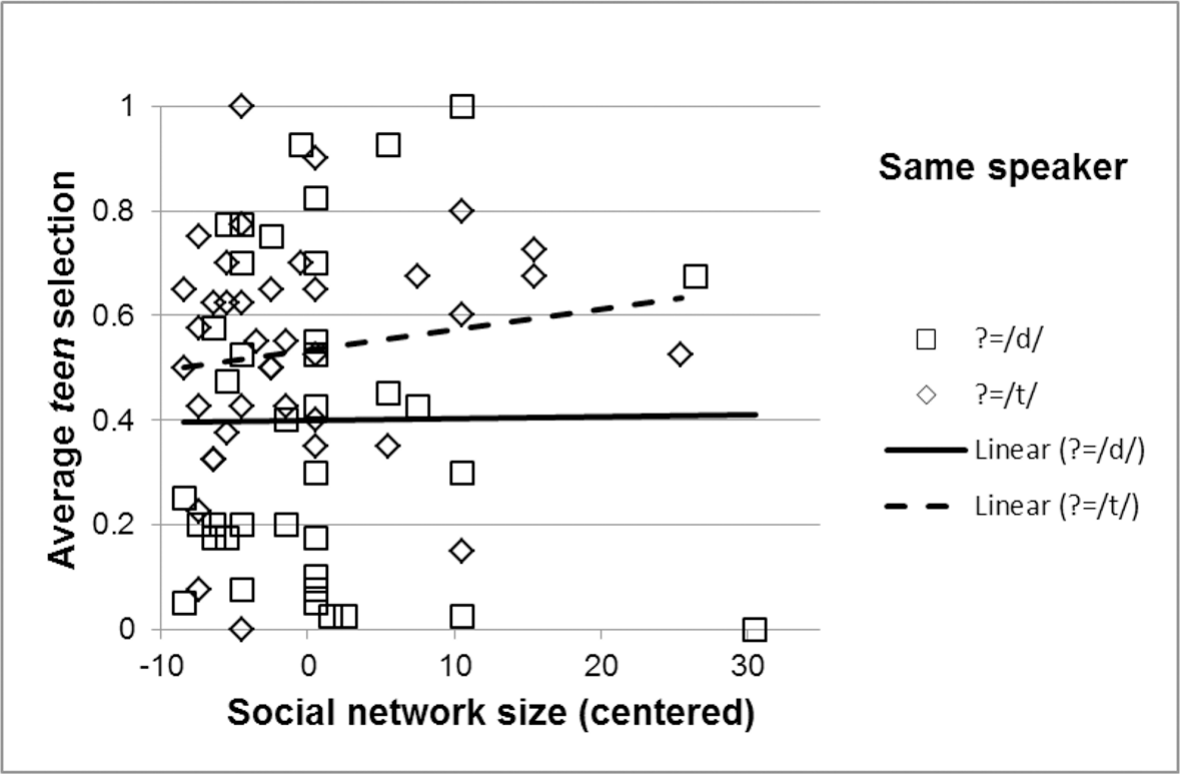
Plot of uncontrolled summary statistics for performance in the Same Speaker condition. Average *teen* selection in the Same Speaker condition as dependent on social Network Size and Audio Condition in exposure. Note that this figure illustrates summary statistics, but that the analysis was not over summary statistics but over the full results. Note also that this figure is only for illustration purposes and the plotted data points show responses without controlling for factors the model controlled for, such as VOT. Similarly to the plotted results of the model in Fig 2, the effect of perceptual learning is manifested in the distance between the line in the manipulated /t/ condition and the line in the manipulated /d/ condition. Greater perceptual learning should lead to more *teen* responses in the manipulated /t/ condition and fewer *teen* responses in the manipulated /d/ condition.

To better understand the nature of the three-way interaction, separate analyses on the Same Speaker and New Speaker conditions were run. A mixed model analysis on the Same Speaker condition revealed an effect of VOT (β = 0.21, SE = 0.01, z = 15.61, p<0.001), such that tokens with longer VOTs were more likely to be classified as *teen*. As before, this indicates that participants performed the task. Results also revealed an effect of Audio Condition (β = 1.38, SE = 0.44, z = 3.11, p = 0.002; See Figs 2 and 3 and S2 Table), indicating that participants in the manipulated /t/ condition classified more tokens as *teen*. This reflects an effect of perceptual learning, as exposure influenced the location of the boundary between the /t/ and /d/ categories, such that listening to a speaker who produces the ambiguous sound for /t/ versus /d/ led to larger /t/ category and smaller /d/ category. Crucially, neither the effect of Social Network Size nor its interaction with Audio Condition reached significance.

**Fig 4:**
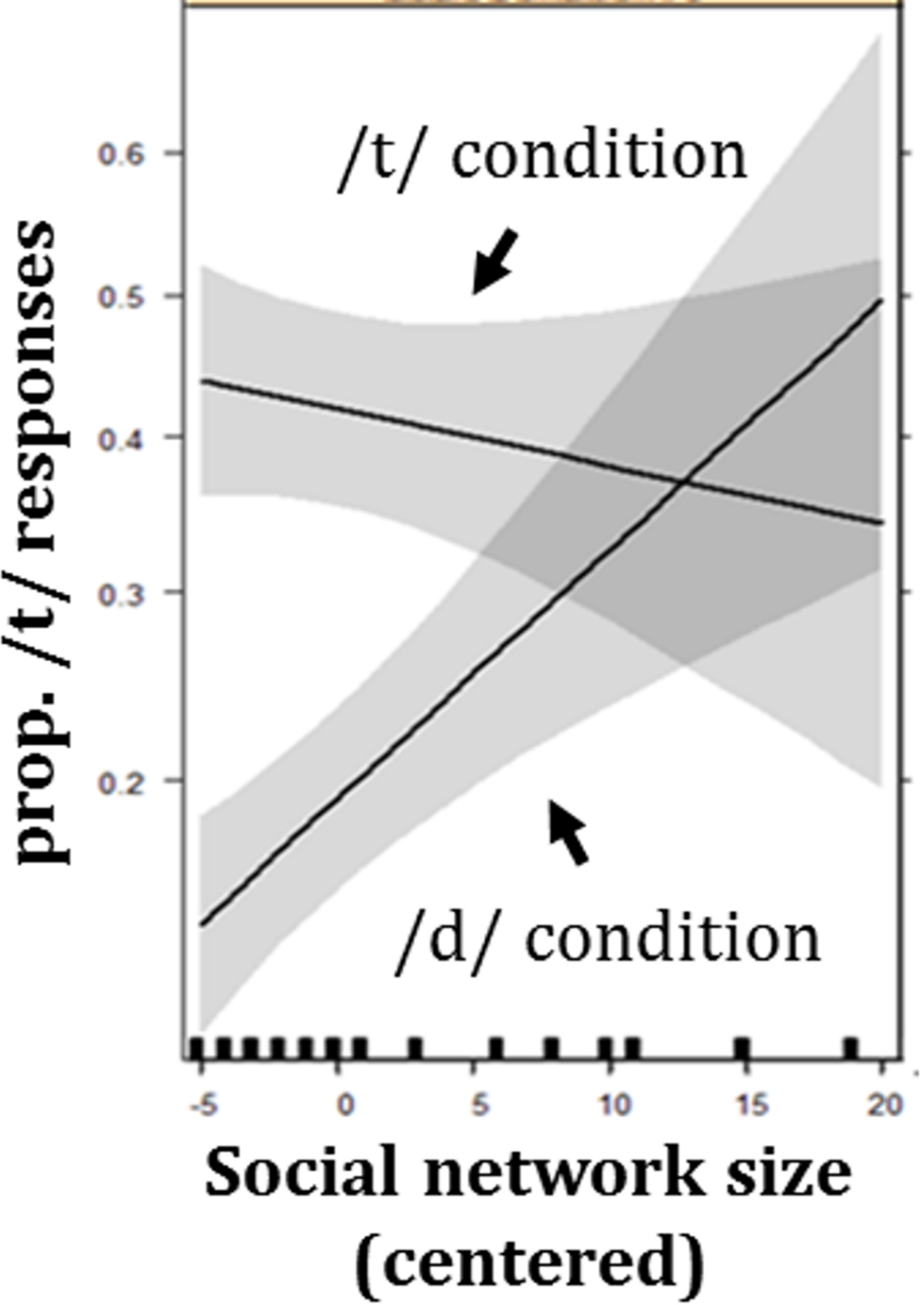
Model’s statistical results for performance in the New Speaker condition. The model’s predictions for the probability of *teen* selection in the New Speaker condition as dependent on Social Network Size and Audio Condition in exposure. The effect of perceptual learning is manifested in the distance between the line in the manipulated /t/ condition and the line in the manipulated /d/ condition, as greater perceptual learning should lead to more *teen* responses in the manipulated /t/ condition and fewer *teen* responses in the manipulated /d/ condition. Gray bands indicate standard errors (67% confidence interval).

**Fig 5:**
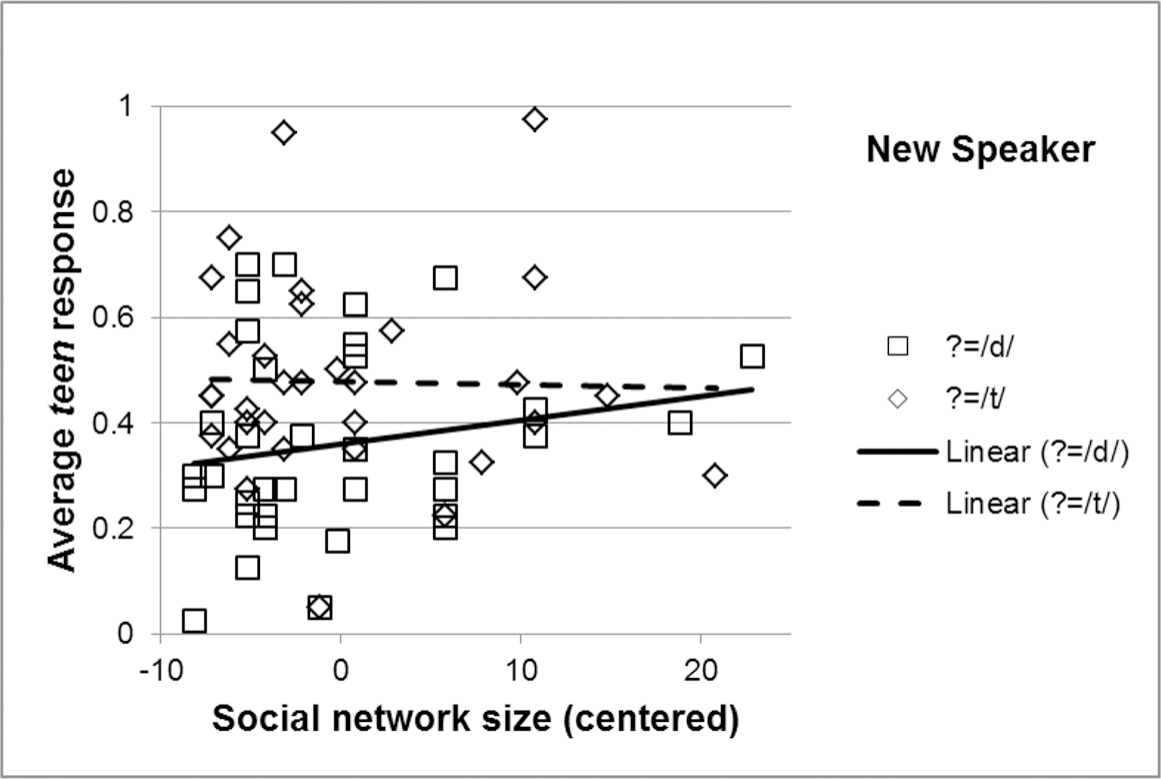
Plot of uncontrolled summary statistics for performance in the New Speaker condition. Average *teen* selection in the New Speaker condition as dependent on Social Network Size and Audio Condition in exposure. Note that this figure illustrates summary statistics, but that the analysis was not over summary statistics but over the full results. Note also that this figure is only for illustration purposes and the plotted data points show responses without controlling for factors the model controlled for, such as VOT. Similarly to the plotted results of the model in Fig 4, the effect of perceptual learning is manifested in the distance between the line in the manipulated /t/ condition and the line in the manipulated /d/ condition, as greater perceptual learning should lead to more *teen* responses in the manipulated /t/ condition and fewer *teen* responses in the manipulated /d/ condition.

In contrast, an analysis of the data in the New Speaker condition revealed not only similar effects of VOT (β = 0.34, SE = 0.02, z = 16.56, p<0.001) and Audio Condition (β = 1.10, SE = 0.36, z = 3.07, p = 0.002), but also an effect of Social Network Size at the reference level of the manipulated /d/ condition, indicating fewer /d/ interpretations, and thus smaller effect of exposure with larger Social Network Size (β = 0.07, SE = 0.04, z = 2.10, p = 0.035; See S3 Table). Results also showed a numeric pattern in line with an interaction between Social Network Size and Audio Condition, but this effect did not reach significance (β = -0.09, SE = 0.05, z = -1.76, p = 0.078). As Figs 4 and 5 show, the difference between the audio conditions was numerically larger for participants with Smaller Social Networks.

It is less clear why the role of Social Network Size was particularly strong in the manipulated /d/ condition. In general, it seems that the perceptual learning effect itself was mostly driven by the manipulated /d/ condition. The VOT values of the continuum were selected such that it would lead to about equal *teen* and *dean* selections in the absence of any exposure. In the manipulated /d/ condition, the proportion of *teen* selection appropriately dropped below 50%. In contrast, the proportion of *teen* selections in the manipulated /t/ condition, in both Speaker conditions, did not rise much above 50%, potentially suggesting that there was no perceptual learning in this condition.

While it is unclear why the manipulated /d/ condition led to greater perceptual learning, the results do show that the perceptual learning effect in the manipulated /d/ condition is, as predicted, largest among individuals with smaller social networks. Furthermore, the lack of any effect of Social Network Size in the Same Speaker condition indicates that this lack of generalization is not due to inability to learn the patterns of the speaker. The similar performance of individuals with different Social Network Sizes in the Same Speaker condition also suggests that the effect that was found in the New Speaker condition is unlikely to be due to other factors that co-vary with Social Network Size and that affect participants' motivation to do the task or their approach to it.

## General discussion

This study shows that individuals with smaller social networks have more malleable representations. Specifically, it shows that those with smaller social networks are more likely to adjust their general representation of phonological categories following exposure to non-normative input. We propose that this greater malleability of representations is due to the fact that when one has only been exposed to few sources, any new source is more informative, and therefore its input is assigned more weight. One potential implication of this finding is that individuals with smaller social networks might be more likely to propagate linguistic innovations, and therefore might play an important role in the diffusion of linguistic changes.

One caveat is that individuals' Social Network Size was not manipulated but exploited the natural variation in circle size. Therefore, theoretically, any effect that was found could be due to causality in the opposite direction or to co-variation with another factor. While this cannot be ruled out completely, the specificity of the effect to the New Speaker condition makes it unlikely to be the case. The lack of an effect of Social Network Size in the Same Speaker condition indicates that people with different Social Network Sizes approached the task similarly and were equally able to learn the pattern. Moreover, the three-way interaction is significant. Furthermore, the lack of an effect of Social Network Size is unlikely to be due to insufficient power, since, if anything, the numeric pattern in the Same Speaker condition goes in the opposite direction to that in the New Speaker condition. While the direction of an effect can be misleading when studies are not sufficiently powered, the significance of the results in the New Speaker condition indicates that there was sufficient power to detect an effect in that case. Furthermore, a similar study that examined the relationship between social network size and the malleability of representations at the lexical level found similar results–social network size had a significant negative effect in the new speaker condition, but a non-significant positive effect in the same speaker condition [28]. As the effect of Social Network Size in the Same Speaker condition was not significant, we do not elaborate its opposite directionality. We would only speculate that it might be the case that having a larger social network improves one’s ability to learn speakers’ phonological patterns. Indeed, in other studies we found that having a larger social network improves certain phonological and semantic abilities (e.g., [23]). This would make it even more remarkable that despite superior learning of the speaker’s speech pattern, participants with larger social networks generalized it less. Importantly, regardless of the underlying cause of the effects that were found, the results of this paper show a relevant and ecologically important pattern. They point to the potentially large role that non-hub members have in propagating changes, leaving open the question regarding how people self-select into different positions in the social network.

Another limitation of the study is that it tested participants’ perception rather than production. The account here assumes that the category boundary that guides our interpretation of input also influences our production, and that we would not produce, for example, /d/s with a VOT that we would interpret as /t/ in perception. This assumption fits with many models of language processing and production, including those that assume that we monitor our production via comprehension [29] or that we use our own production system to predict others’ speech during language processing [30]. Nonetheless, it would be important to follow up on these studies with an experiment that examines production.

These results relate to the threshold problem in language change—the puzzle regarding how rare forms, especially ones that do not have any obvious advantage, get adopted and diffuse through the network. While Social Network Size cannot account for such diffusion on its own, combining small Social Network Size with other assumptions, such as assigning higher weight to recent input, could explain how rare variants can overcome the threshold problem. Other factors, such as the social status of the speaker, might play a role as well and help boost learning further in some cases [22].

On the face of it, one may wonder whether greater malleability is sufficient to allow people with smaller social networks to play an important role in linguistic diffusion. In particular, people might be less likely to be influenced by people with smaller social networks, minimizing their ability to propagate rare variants. For example, [31] suggested that the influence of individuals is directly proportional to the number of contacts they have. At the same time, others have shown that it is those with weak ties that are important for diffusion [32–35]. They argued that members with weak ties are more likely to have access to other types of information, and are less constrained by the group's norms [33, 36]. Furthermore, studies of diffusion of information, trends, and even hysterical medical symptoms, have shown that weak ties are often crucial for the diffusion of information and trends [33]. In fact, the elimination of bridges, which are weak ties that uniquely connect individuals, is much more deleterious for diffusion than the elimination of strong ties. While having a smaller social network is not equivalent to being a weak tie, these previous findings show that individuals learn not only from central members, but also from peripheral ones. Therefore, it is possible that individuals would be influenced by people with smaller social networks despite the fact that these individuals are unlikely to be central members in the community. Indeed, some have even argued that some risky behaviors are more likely to first spread via peripheral members, as central members need to guard their reputation more. For similar reasons, it has even been argued that social action is more likely to be organized via weak ties than via strong ties [37].

It is important to note that our studies focused on susceptibility to novel input that carries no social meaning. That is, intermediate VOTs are not associated with any social marker or sub-group. Furthermore, the deviation of the input from the norm was not even noticeable to our participants. While many linguistic changes are of this type, some innovations are highly noticeable and associated with different social identities. The results of our studies are most relevant to situations where this is not the case. The number of sources and the statistical weighting are likely to be more pertinent when an explicit wish to project a certain identity and avoid another are irrelevant, and the learning is mostly implicit. It is an interesting question how social network size interacts with social factors in cases where linguistic innovations are more marked and socially motivated.

To conclude, this study shows that individuals' social network size influences how malleable their linguistic representations are. It thus suggests that individuals with small social networks might play an important role in the propagation of linguistic innovations.

## Supporting information

S1 Table Main table of results. Table of results of the main analysis of Experiment 1.
(DOCX)

S2 Table Results of Same Speaker condition. Table of results of the analysis in the Same Speaker condition.
(DOCX)

S3 Table Results of New Speaker condition. Table of results of the analysis in the New Speaker condition.
(DOCX)

S1 Text Alternative statistical analyses. Information about alternative statistical analyses to those reported in the paper.
(DOCX)

S2 Text Social network questionnaire. The full social network questionnaire used in the experiment.
(DOCX)

S1 File Raw data. The raw results of the experiment.
(CSV)

## References

[1] Goodman J C, Dale PS, Li P. Does frequency count? Parental input and the acquisition of vocabulary. J Child Lang. 2008; 35: 3: 515–531. doi: 10.1017/S0305000907008641 18588713 10.1017/S0305000907008641

[2] Maye J, Werker J F, Gerken L. Infant sensitivity to distributional information can affect phonetic discrimination. Cognition.2002; 82: 3: B101–B111. 11747867 10.1016/s0010-0277(01)00157-3

[3] Rott S. The effect of exposure frequency on intermediate language learners’ incidental vocabulary acquisition and retention through reading. Stud Second Lang Acquis. 1999; 21: 4: 589–619.

[4] Saffran JR, Aslin RN. Newport EL. Statistical learning by 8-months-old infants. Science. 1996; 274: 1926–1928. 8943209 10.1126/science.274.5294.1926

[5] Brysbaert M, Buchmeier M. Conrad M: Jacobs AM: Bülte J: Bühl A. The word frequency effect: A review of recent developments and implications for the choice of frequency estimates in German. Exp Psychol. 2011; 58: 412–424. doi: 10.1027/1618-3169/a000123 21768069 10.1027/1618-3169/a000123

[6] Harrington J, Palethorpe S. Watson CI. Does the Queen speak the Queen's English?. Nature. 2000; 408: 6815: 927–928. 11140668 10.1038/35050160

[7] Kraljic T, Samuel AG. Perceptual adjustments to multiple speakers. J Mem Lang. 2007; 56: 1–15

[8] Norris D, McQueen JM. Cutler A. Perceptual learning in speech. Cogn Psychol. 2003; 47: 2: 204–238. 12948518 10.1016/s0010-0285(03)00006-9

[9] Cook V., editor. Effects of the second language on the first. Clevedon: UK: Multilingual Matters 2003.

[10] Flege JE. The production of “new” and “similar” phones in a foreign language: Evidence for the effect of equivalence classification. J Phon. 1987; 15: 1: 47–65.

[11] Lev-Ari S, Keysar B. Executive control influences linguistic representations. Mem Cognit. 2014; 42: 247–263. doi: 10.3758/s13421-013-0352-3 23928859 10.3758/s13421-013-0352-3

[12] Lev-Ari S, Peperkamp S. Low inhibitory skill leads to non-native perception and production in bilinguals’ native language. J Phon. 2013; 41: 320–331.

[13] Pavlenko A, Jarvis S. Bidirectional transfer. Appl Linguist. 2002; 23: 2: 190–214.

[14] Sankoff G. Longitudinal Studies In Bayley R. Cameron R. Lucas C., editors. Oxford Handbook of Sociolinguistics. Oxford University Press; 2013 261–279.

[15] Goldinger SD. Echoes of echoes? An episodic theory of lexical access.Psychol Rev. 1998; 105:251–79. 9577239 10.1037/0033-295x.105.2.251

[16] Johnson K. Speech perception without speaker normalization In: K Johnson, Mullennix JW, editors. Talker variability in speech processing. San Diego: Academic Press; 1997 145–65.

[17] Kleinschmidt DF, Jaeger TF. Robust speech perception: Recognize the familiar: generalize to the similar: and adapt to the novel. Psychol Rev. 2015; 122: 2: 148 doi: 10.1037/a0038695 25844873 10.1037/a0038695 PMC4744792

[18] Haun DB, Rekers Y, Tomasello M. Majority-biased transmission in chimpanzees and human children: but not orangutans. Curr Biol. 2012;22: 8: 727–731. doi: 10.1016/j.cub.2012.03.006 22503497 10.1016/j.cub.2012.03.006

[19] Corriveau KH, Fusaro M, Harris PL. Going with the flow: preschoolers prefer nondissenters as informants. Psychol Sci. 2009; 20: 372–377. doi: 10.1111/j.1467-9280.2009.02291.x 19207691 10.1111/j.1467-9280.2009.02291.x

[20] Herrmann P, Legare CH, Harris PL: Whitehouse H. Stick to the script: The effect of witnessing multiple actors on children's imitation. Cognition. 2013; 129: 536–543. doi: 10.1016/j.cognition.2013.08.010 24045001 10.1016/j.cognition.2013.08.010

[21] Schillaci RS, Kelemen D. Children's Conformity When Acquiring Novel Conventions: The Case of Artifacts. J Cogn Dev. 2013; 15: 569–583.

[22] Nettle D. Using Social Impact Theory to simulate language change. Lingua. 1999; 118: 2–3: 95–117.

[23] Lev-Ari S. How the size of our social network influences our semantic skills. Cogn Sci. 2016; 40: 2050–2064. doi: 10.1111/cogs.12317 26515021 10.1111/cogs.12317

[24] Lev-Ari S, Shao Z. How social network heterogeneity facilitates lexical access and lexical prediction. Mem Cognit. 2017; 45: 3: 528–538. doi: 10.3758/s13421-016-0675-y 27896710 10.3758/s13421-016-0675-y PMC5368194

[25] Hoaglin DC, Welsch RE. The hat matrix in regression and ANOVA. The American Statisticians. 1978; 32: 17–22.

[26] R Core Team. R: A language and environment for statistical computing. R Foundation for Statistical Computing, Vienna, Austria Available online at: https://www.R-project.org/. 2016.

[27] Bates D, Maechler M, Bolker B, Walker S. Fitting Linear Mixed-Effects Models Using lme4. J Stat Softw. 2015; 67: 1: 1–48.

[28] Lev-Ari S. People with smaller social networks are more influenced by new speakers. In: Proceedings of the 30th CUNY Conference on Human Sentence Processing: Cambridge MA: USA. 2017.

[29] Levelt WJM. Monitoring and Self-Repair in Speech. Cognition. 1983; 14: 1: 41–104. 6685011 10.1016/0010-0277(83)90026-4

[30] Pickering MJ, Garrod S. Do people use language production to make predictions during comprehension?. Trends Cogn Sci. 2007; 11: 3: 105–110. doi: 10.1016/j.tics.2006.12.002 17254833 10.1016/j.tics.2006.12.002

[31] Fagyal Z, Swarup S, Escobar AM, Gasser L, Lakkaraju K. Centers and peripheries: Network roles in language change. Lingua. 2010; 120: 8: 2061–2079.

[32] Bakshy E, Rosenn I, Marlow C, Adamic L. The role of social networks in information diffusion. In: Proceedings of the 21st international conference on World Wide Web; 2012. pp. 519–528.

[33] Granovetter MS. The strength of weak ties. Am J Sociol. 1973; 78: 6: 1360–1380.

[34] Mühlenbernd R, Franke M. Signaling conventions: who learns what where and when in a social network. In: Proceedings of EvoLang IX; 2012. pp. 242–249.

[35] Weimann G. On the importance of marginality: One more step into the two-step flow of communication. Am Sociol Rev. 1982; 764–773.

[36] Milroy J, Milroy L. Linguistic change: social network and speaker innovation. J Linguist. 1985 21: 339–384.

[37] Granovetter M. The strength of weak ties: A network theory revisited. Sociological theory.1983; 1: 1: 201–233.

