# Supplementary material for: Talking to fewer people leads to having more malleable linguistic representations
Source: PLoS One. 2017 Aug 24;12(8):e0183593. doi: 10.1371/journal.pone.0183593 (PMC5570344; doi:10.1371/journal.pone.0183593)
Supplement: S1 Table — Table of results of the main analysis of Experiment 1. (DOCX) [file pone.0183593.s001.docx]

|  | β | SE | z | p-value |
| --- | --- | --- | --- | --- |
| (intercept) | -0.96 | 0.30 | -3.17 | 0.002 |
| VOT | 0.27 | 0.01 | 21.79 | < 2e-16 |
| Audio Condition (/t/) | 1.06 | 0.42 | 2.51 | 0.012 |
| Speaker (same) | -0.58 | 0.43 | -1.36 | 0.175 |
| Network Size | 0.08 | 0.04 | 1.90 | 0.057 |
| Speaker X Audio Condition | 0.38 | 0.58 | 0.65 | 0.515 |
| Audio Condition x Network Size | -0.09 | 0.06 | -1.55 | 0.121 |
| Speaker X Interlocutors | -0.1 | 0.05 | -1.80 | 0.073 |
| Speaker X Audio Condition x Network Size | 0.17 | 0.08 | 2.10 | 0.036 |
