# Supplementary material for: Talking to fewer people leads to having more malleable linguistic representations
Source: PLoS One. 2017 Aug 24;12(8):e0183593. doi: 10.1371/journal.pone.0183593 (PMC5570344; doi:10.1371/journal.pone.0183593)
Supplement: S2 Table — Table of results of the analysis in the Same Speaker condition. (DOCX) [file pone.0183593.s002.docx]

|  | β | SE | z | p-value |
| --- | --- | --- | --- | --- |
| (intercept) | -1.09 | 0.33 | -3.32 | <0.001 |
| VOT | 0.21 | 0.01 | 15.61 | < 2e-16 |
| Audio Condition (/t/) | 1.38 | 0.44 | 3.11 | 0.002 |
| Network Size | -0.02 | 0.04 | -0.51 | 0.611 |
| Audio Condition x Network Size | 0.07 | 0.06 | 1.20 | 0.232 |
