# Supplementary material for: Talking to fewer people leads to having more malleable linguistic representations
Source: PLoS One. 2017 Aug 24;12(8):e0183593. doi: 10.1371/journal.pone.0183593 (PMC5570344; doi:10.1371/journal.pone.0183593)
Supplement: S3 Table — Table of results of the analysis in the New Speaker condition. (DOCX) [file pone.0183593.s003.docx]

|  | β | SE | z | p-value |
| --- | --- | --- | --- | --- |
| (intercept) | -1.43 | 0.27 | -5.37 | 7.93e-08 |
| VOT | 0.34 | 0.02 | 16.56 | < 2e-16 |
| Audio Condition (/t/) | 1.10 | 0.36 | 3.07 | 0.002 |
| Network Size | 0.07 | 0.04 | 2.10 | 0.035 |
| Audio Condition x Network Size | -0.09 | 0.05 | -1.76 | 0.078 |
