# Supplementary material for: Talking to fewer people leads to having more malleable linguistic representations
Source: PLoS One. 2017 Aug 24;12(8):e0183593. doi: 10.1371/journal.pone.0183593 (PMC5570344; doi:10.1371/journal.pone.0183593)
Supplement: S1 Text — Information about alternative statistical analyses to those reported in the paper. (DOCX) [file pone.0183593.s004.docx]

**Alternative statistical analyses**

1. Analyses that do not exclude the participants whose Social Network Size was 4 and 8 standard deviations from the mean show very different results from those reported in the paper. Comparing results with and without a particular participant or observation is in fact the test for undue influence, often called Cook’s Distance [1]. Analyses whose results depend on including a specific data point or individual are considered unreliable.
2. Analyzing the data with the untrimmed estimates of Social Network Size leads to the same pattern of results as in the analysis reported in the paper, but the triple interaction does not reach conventional level of significance (β=0.13263, SE=0.07405, z=1.791, p=0.073).

**References**

[1] Cook RD. Detection of influential observation in linear regression. Technometrics. 1977; 19: 1: 15-18.
