# Supplementary material for: Talking to fewer people leads to having more malleable linguistic representations
Source: PLoS One. 2017 Aug 24;12(8):e0183593. doi: 10.1371/journal.pone.0183593 (PMC5570344; doi:10.1371/journal.pone.0183593)
Supplement: S2 Text — The full social network questionnaire used in the experiment. (DOCX) [file pone.0183593.s005.docx]

**Social network questionnaire**

In this questionnaire we would like to gather information about your linguistic interactions. We realize that some of the estimates are difficult to make. Please do your best and be as accurate as possible.

Important: When providing estimates for your exposure in a week, keep in mind that your habits may vary considerably depending on the day of the week (e.g., weekday vs. weekend). Please be as accurate as possible and do not simply multiply your estimate for one day by 7.

1) How old are you?


2) With how many people do you converse orally **in a typical week**? (Please only include people with whom you regularly talk for longer than 5 minutes)


3) How many hours do you usually spend on conversing orally with people **in a typical week**?


4) How are the people you converse with **in a typical week** related to you (e.g. friend, colleague, family, service person, neighbor etc.)? Please indicate the relations with an estimate of how many people fall there (e.g., 3 relatives, 10 colleagues etc.).

| **Number of people** | **Relation to you** |
| --- | --- |
|  |  |
|  |  |
|  |  |
|  |  |
|  |  |
|  |  |
|  |  |
|  |  |

5a) Please state the age range of the people with whom you regularly converse **in a typical week** from the youngest person to the oldest person (e.g. 21-60 years). Only include those above the age of 12.


5b) What is the main age group with which you interact **in a typical week**? (e.g. if you mainly interact with your friends who are between 20-25 years old (20 people) and you also interact with your parents who are 50 years old (2 people), the most common age group would be 20-25 years. If there is not one main age group, write "varied")


6a) Please indicate what are the highest and lowest levels of education of the people you interact with **in a typical week** (e.g. high school diploma - PhD). Only include the level of education of people above the age of 22.


6b) What is the most common education level among those people? (e.g. if you mainly interact with college graduates, but you also occasionally converse with people who did not go to college, the most common education level would be Bachelor degree. If there is not one dominant educational level, write "varied")


7) What proportion of the people you converse with in a typical week know one another?
